# The Bacterial DNA Binding Protein MatP Involved in Linking the Nucleoid Terminal Domain to the Divisome at Midcell Interacts with Lipid Membranes

**DOI:** 10.1128/mBio.00376-19

**Published:** 2019-05-28

**Authors:** Begoña Monterroso, Silvia Zorrilla, Marta Sobrinos-Sanguino, Miguel Ángel Robles-Ramos, Carlos Alfonso, Bill Söderström, Nils Y. Meiresonne, Jolanda Verheul, Tanneke den Blaauwen, Germán Rivas

**Affiliations:** a Centro de Investigaciones Biológicas, Consejo Superior de Investigaciones Científicas (CSIC), Madrid, Spain; b Structural Cellular Biology Unit, Okinawa Institute of Science and Technology, Okinawa, Japan; c Bacterial Cell Biology & Physiology, Swammerdam Institute for Life Sciences, University of Amsterdam, Amsterdam, the Netherlands; Duke University School of Medicine

**Keywords:** DNA binding proteins, bacterial division, biochemical reconstruction, division site selection, protein-membrane interaction

## Abstract

Division ring formation at midcell is controlled by various mechanisms in Escherichia coli, one of them being the linkage between the chromosomal Ter macrodomain and the Z-ring mediated by MatP, a DNA binding protein that organizes this macrodomain and contributes to the prevention of premature chromosome segregation. Here we show that, during cell division, just before splitting the daughter cells, MatP seems to localize close to the cytoplasmic membrane, suggesting that this protein might interact with lipids. To test this hypothesis, we investigated MatP interaction with lipids *in vitro*. We found that, when encapsulated inside vesicles and microdroplets generated by microfluidics, MatP accumulates at phospholipid bilayers and monolayers matching the lipid composition in the E. coli inner membrane. MatP binding to lipids was independently confirmed using lipid-coated microbeads and biolayer interferometry assays, which suggested that the recognition is mainly hydrophobic. Interaction of MatP with the lipid membranes also occurs in the presence of the DNA sequences specifically targeted by the protein, but there is no evidence of ternary membrane/protein/DNA complexes. We propose that the association of MatP with lipids may modulate its spatiotemporal localization and its recognition of other ligands.

## Abstract (precis)

The division of an E. coli cell into two daughter cells with equal genomic information and similar size requires duplication and segregation of the chromosome and subsequent scission of the envelope by a protein ring, the Z-ring. MatP is a DNA binding protein that contributes both to the positioning of the Z-ring at midcell and the temporal control of nucleoid segregation. Our integrated *in vivo* and *in vitro* analysis provides evidence that MatP can interact with lipid membranes reproducing the phospholipid mixture in the E. coli inner membrane, without concomitant recruitment of the short DNA sequences specifically targeted by MatP. This observation strongly suggests that the membrane may play a role in the regulation of the function and localization of MatP, which could be relevant for the coordination of the two fundamental processes in which this protein participates, nucleoid segregation and cell division.

## INTRODUCTION

Bacterial division is achieved through the assembly of a protein machinery into a membrane-anchored ring that splits the cell, generating two daughter cells with equal genomic information (1). The scaffold for the involved proteins is the self-assembling protein FtsZ. The need of a precise localization of this Z-ring in the middle of the cell is fulfilled by different mechanisms evolved in bacteria, the canonical ones being the Min system and nucleoid occlusion (2). An additional mechanism contributing to Z-ring positioning is the linkage between the Ter macrodomain of the chromosome and the Z-ring (Ter linkage) (3). While the two first systems exert their action through blockage of productive FtsZ assembly at certain locations, namely the vicinity of the nucleoid and the cell poles, the last one is a positive mechanism promoting assembly of the division machinery nearby the replication terminus region of the chromosome (4).

The Ter linkage consists of three proteins, MatP, ZapB, and ZapA, which form a complex that links the chromosome to the Z-ring. MatP, a DNA binding protein, was identified by Mercier and coworkers (5), who showed that it is the main organizer of the Ter macrodomain of the chromosome, preventing its premature segregation through specific interaction with a short palindromic DNA sequence (*matS*) repeated 23 times within this macrodomain. There are no *matS* sequences outside the Ter macrodomain, which is in turn devoid of the sequences targeted by SlmA, the other DNA binding protein avoiding, through nucleoid occlusion, aberrant Z-ring positioning (6). It was recently found that, upon binding to the *matS* sites, MatP displaces MukBEF from the Ter domain (7), promoting the formation of a unique chromosomal region. The Ter domain progressively shifts toward the cell center along the cell cycle (8) and by binding to ZapB remains localized at midcell during division in slowly growing cells (9, 10). Through the last cell division stage, the Ter macrodomain is segregated into each daughter cell while they separate. The cell division protein FtsK forms probably at this stage a hexameric DNA translocase that moves about 400 bp toward the *dif* sites close to the terminus of the chromosome while displacing MatP from its *matS* sites (11, 12) to assist in the segregation of the termini. The molecular mechanisms by which this last step of chromosome segregation and daughter cell separation are coordinated remain largely unknown.

In this work, we observed that MatP moves away from the division site near the end of the cell division cycle, leaving a still intact divisome, including ZapB at midcell. Indeed, also the colocalization with the nucleoids seemed to be at least partly lost, and MatP was often observed close to the cytoplasmic membrane. On the basis of these findings, we postulated that MatP could bind to lipid membranes and verified this hypothesis *in vitro*, through reconstruction of the purified protein inside microfluidics microdroplets and giant unilamellar vesicles (GUVs), in the absence and presence of a *matS* oligonucleotide. Parallel experiments based on complementary biochemical approaches further supported the interaction of MatP with lipids. We propose that the membrane binding of MatP serves to free the *matS* sites close to the *dif* site that is needed by FtsK to help the segregation of the termini into the two daughter cells.

## RESULTS

### MatP localizes between the nucleoid and ZapB at the end of the cell division cycle.

To investigate what the exact sequence of events is during the process of cell division, we previously analyzed the localization of a large number of cell division proteins in steady-state slowly growing cells (13, 14). The advantage of slowly growing cells is that they do not have multiple replication forks—at least during the major part of their division cycle. When Escherichia coli cells are grown to steady state, their length correlates well with the cell division cycle age. We have now investigated, as part of the proteins that are involved in the coupling of cell division and chromosome segregation, the localization of the nucleoids in relation to that of MatP and the protein complex responsible for division (divisome). MG1655 cells expressing MatP-mCherry (MatP-mCh) (15) from the original locus in the chromosome were grown in minimal medium to steady-state. In these cells, the localization of its divisome partner, ZapB, and the divisome protein, FtsN, which marks the presence of a complete division machinery, was determined by immunolabeling.

**FIG 1:**
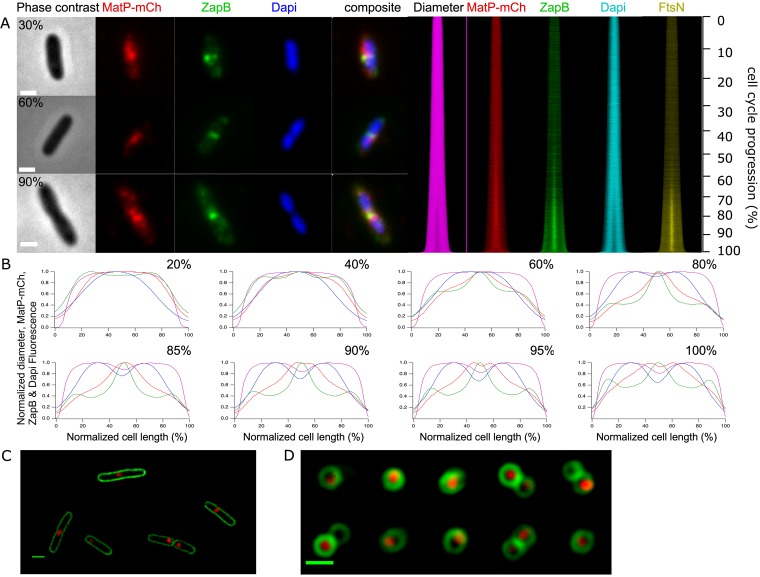
Localization of MatP as a function of the cell division cycle. (A) A representative example of a cell of age classes: 30%, 60%, and 90% from top to bottom. From left to right, phase-contrast and fluorescence images of MatP-mCh, immunolabeled ZapB, and DAPI-stained nucleoids are shown. The map of the profiles shows in the same order the diameter determined in the phase-contrast images and fluorescence as a function of cell length. The numbers on the right show the relationship between length and cell division cycle age. (B) Peak normalized average profiles from the maps of the diameter and fluorescence were plotted against the normalized cell length in age bins of 0 to 20%, 20 to 40%, 40 to 60%, 60 to 80%, 80 to 85%, 85 to 90%, 90 to 95%, and 95 to 100%. The age class with the smallest number of cells (i.e., 95 to 100%) still contains 592 cells. In total, 16,796 cells were analyzed. (C) SIM images of live MG1655 *matP-mCh*::*kan* cells transformed with plasmid pXL28, which expresses the integral membrane protein fusion mNeonGreen-(GGS)_2_-GlpT. The cells had been grown in Gb4 minimal medium at 28°C and induced for 2 mass doublings with 30 μM IPTG. (D) SIM images of upright cells grown as in panel C. The 10 cells shown are all from a single image without selection and were grouped to reduce the figure size. All scale bars equal 2 μm.

To be able to dissect what happens to the localization of these three proteins and the nucleoid (stained by DAPI [4′,6-diamidino-2-phenylindole]), more than 16,000 cells were imaged and analyzed. MatP-mCh and ZapB colocalize during most of the cell division cycle, and FtsN arrives later at midcell (Fig. 1A) as described previously (8, 13, 15). The concentration of MatP is constant during the cell cycle (see Fig. S1A in the supplemental material). The number of MatP dimers (species assumed based on previous structural *in vitro* data [15]) per average cell in minimal medium was determined to be 180 (16). Using this number and the determined extra fluorescence at midcell (FCPlus [14]), the number of MatP dimers was calculated to be 60 in the foci at midcell at 80% of the cell division cycle age (Fig. S1B). MatP localizes in young cells as a diffuse focus, which moves toward the cells center during the cell division cycle, where it forms a more distinct concentrated focus (Fig. 1A, B, and Fig. S1C). When determining the position of the brightest pixel in the MatP foci, they seem to localize consistently close to the length axis of the cell (Fig. S1D), as was reported (17). However, after 90% of the cell division cycle, MatP-mCh moves away from the divisome, whereas ZapB and FtsN remain almost until the cells are completely divided (Fig. 1A). Interestingly, inspection of the deeply constricting cells suggested that MatP is not following the nucleoid that is segregating but remains between ZapB and the nucleoid. This suggests that at least part of the MatP protein is not binding to the Ter domain any longer and also not binding to ZapB.

10.1128/mBio.00376-19.2 FIG S1 Concentration of MatP as a function of the cell division cycle. (A) The concentration of MatP (plotted as molecules per μm^3^) is constant during the cell division cycle (plotted as normalized cell division cycle age).The average number of MatP dimers in the minimal medium-grown cells was assumed to be 180 based on previous work (16). (B) FCPlus or the extra number of MatP molecules at midcell (area of 0.4 μm × the width of the cell) compared to the number of molecules in the rest of the cell plotted as a function of the normalized cell division cycle age (%). The markers are 5% age bins, and the error bars give the 95% confidence interval. (C) Average fluorescence profile of the cells (with the profile of MatP-mCh flipped so that all cells are oriented left with the pole with highest fluorescence intensity) plotted against their normalized cell length (%). This shows the movement of the MatP focus from new pole to midcell as a function of cell division cycle age in bins of 10%. (D) Distance of the brightest pixel in MatP foci from the length axis of the cells plotted as function of normalized cell division cycle age. The number of analyzed cells was >16,000. In panels A, B, and D, gray dots are the values for the individual cells. Download FIG S1, TIF file, 2.1 MB. Copyright © 2019 Monterroso et al. 2019 Monterroso et al. This content is distributed under the terms of the Creative Commons Attribution 4.0 International license.

Since we observed the signal of MatP was often close to the membrane of the new poles, we wondered whether MatP might bind lipids, like was observed for other proteins binding to the chromosome, such as the Noc protein (18). To determine whether MatP-mCh colocalized with the cytoplasmic membrane, we transformed MG1655::MatP-mCh with plasmid pXL28, which expresses the integral membrane protein fusion mNeonGreen-(GGS)_2_-GlpT (mNG-GlpT [19]). Cells were grown to steady state, and the colocalization of MatP and GlpT was determined by the colocalization of the fluorescence of both proteins using the Pearson coefficient (20) as a function of the cell division cycle (see Fig. S2A in the supplemental material). The same strain without plasmid was used to determine the amount of overlap of the mCh channel due to autofluorescence. The Pearson coefficient did increase from 0.18 ± 0.13 in cells without the membrane-staining mNG-GlpT fusion to 0.32 ± 0.13 in cells that did express the protein, indicating some overlap. Because MatP-mCh consisted of one or two foci per cell and the mNG-GlpT was distributed evenly in the cell membrane, not a large overlap was to be expected, and no striking difference in the very old cells was observed (Fig. S2A).

10.1128/mBio.00376-19.3 FIG S2 Pearson’s coefficient for MatP-mCh and mNG-GlpT fluorescence and profiles of individual cells. (A) MG1655 *matP-mCh*::*kan* transformed with plasmid pXL28, which expresses the integral membrane protein fusion mNeonGreen-(GGS)2-GlpT, was grown in Gb4 minimal medium at 28°C and induced for 2 mass doublings with 15 μM IPTG. The cells were concentrated and imaged live by wide-field epifluorescence microscopy. The Pearson’s coefficient is plotted against the normalized cell division cycle age. The markers are the average of the 5% age bin, and the error bars are the 95% confidence interval. Black indicates cells that did not express GlpT (autofluorescence), and red indicates GlpT-expressing cells. The gray (*n* > 4,000) and light-red (*n* > 5,200) dots are the data from the individual cells depicting the absence and presence of GlpT, respectively. Cell age is calculated from its contour area with respect to the entire population, which was growing at steady state. (B) Normalized intensity of a MatP-mCh profile (red) and an mNG-GlpT profile (green) as a function of the pixel positions of the 10 cells shown in Fig. 1D, counting from 1 to 5 in the top row and from 6 to 10 in the bottom row. The profile was determined along a line that was drawn through the center of the mCh-MatP focus (an example is given in the inset image). For most MatP-mCh foci, the center of fluorescence does not overlap the center of fluorescence of the membrane-staining mNG-GlpT. Download FIG S2, TIF file, 1.9 MB. Copyright © 2019 Monterroso et al. 2019 Monterroso et al. This content is distributed under the terms of the Creative Commons Attribution 4.0 International license.

The increase found in the Pearson coefficient could be attributed to the interaction with the membrane of MatP-mCh molecules bound to the DNA or of MatP-mCh molecules detached from the nucleoid. Wide-field fluorescence microscopy could not discriminate between these two options. Therefore, we used structured illumination microscopy (SIM) of cells (Fig. 1C) immobilized in an upright position (Fig. 1D) using an agar pad with a range of micrometer-sized holes and looked at the colocalization of MatP and GlpT. A collection of cells taken from one image (no selection) is shown in Fig. 1D. Many foci localized in the middle of the circumference of the cell short axis, and some colocalization of MatP and the membrane was observed, reinforcing the idea that MatP could interact with lipids. The resolution of the microscope and the intensity of the mCherry signal were not sufficient to discriminate binding of single mCherry molecules to the membrane. Therefore, we decided to investigate the membrane binding of MatP further *in vitro*.

### MatP accumulates at the lipid boundaries of microdroplets and vesicles.

**FIG 2:**
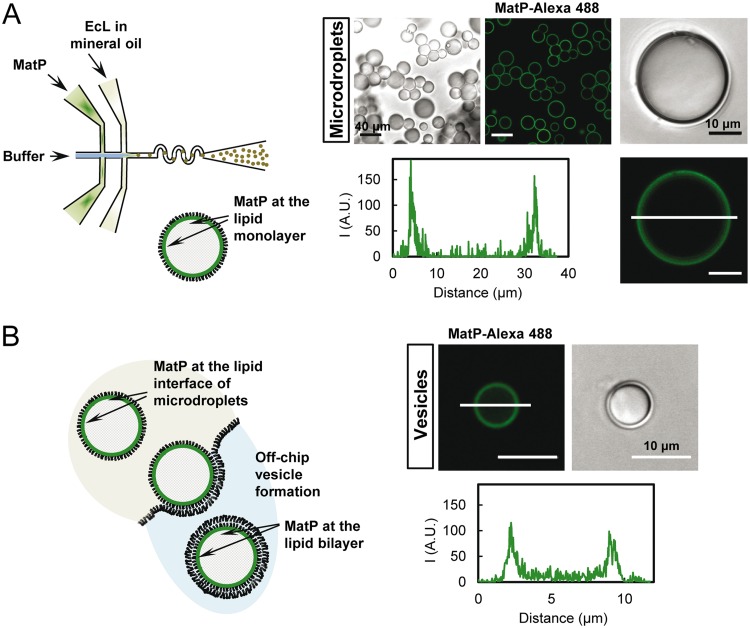
Microfluidic encapsulation of MatP inside microdroplets stabilized by the E. coli lipid mixture and GUVs formed from them. (A) Scheme of the encapsulation setup and of the distribution of species within the droplet (left). Representative confocal and transmitted images of the microdroplets containing MatP (3.5 μM), and intensity profile corresponding to the green channel (MatP-Alexa 488 [1 μM]), obtained across the line as drawn in the image (right). (B) Illustration of the step determining vesicle formation from the droplets with MatP and of the distribution of species within the GUVs (left). Shown are representative confocal and transmitted images of GUVs and an intensity profile corresponding to the green channel (MatP-Alexa 488), obtained across the line as drawn in the image (right). Vesicles contained 150 g/liter Ficoll.

With the aim of investigating whether MatP had lipid affinity, we encapsulated the protein, using microfluidics-based technology, inside microdroplets as cell mimic systems surrounded by a lipid boundary resembling that of the E. coli inner membrane. MatP (with a trace amount of MatP-Alexa 488) was included in one of the aqueous streams, the other one being buffer (Fig. 2A). Microdroplets were formed when the aqueous solutions met the continuous phase, constituted by the E. coli lipids dispersed in mineral oil, at the production junction of the microchip. Interestingly, according to the confocal microscopy images of the samples, MatP was mostly located at the lipid interface inside the microdroplets, as reflected by the intensity profiles (Fig. 2A). Interaction of the protein with lipids was also found when the solution encapsulated inside microdroplets contained crowding agents like Ficoll or dextran together with MatP (see Fig. S3A and B in the supplemental material). Encapsulation of free Alexa 488 dye (Fig. S3C) or of labeled proteins that do not significantly interact with lipids (see, for example, reference 21) allowed the discarding of artifactual binding triggered by the dye and/or the encapsulation method.

10.1128/mBio.00376-19.4 FIG S3 Representative confocal and transmitted images of microdroplets generated by microfluidics containing crowding agents and encapsulation of free dye. (A and B) MatP (3.5 μM, with a trace concentration of 1 μM MatP-Alexa 488) with 150 g/liter Ficoll (A) or dextran (B). The intensity profiles below correspond to the green channel, obtained across the line drawn in the image. (C) Representative images of microdroplets generated by manual bulk emulsion, as previously described (48), with free 1 μM Alexa Fluor 488 dye. Download FIG S3, TIF file, 1.4 MB. Copyright © 2019 Monterroso et al. 2019 Monterroso et al. This content is distributed under the terms of the Creative Commons Attribution 4.0 International license.

After this observation, we wanted to study whether MatP interaction with the lipids also occurred when the lipid boundary was a bilayer, which provides a better cell-like system, instead of the monolayer surrounding the microdroplets. For this purpose, the microdroplets obtained by microfluidics were converted into GUVs, using a procedure based on the droplet transfer method (22), as previously described (21). The droplets acquired the bilayer upon transition from an oil phase to an aqueous solution through an interface coated with oriented lipids (Fig. 2B). The crowding agent Ficoll was encapsulated alongside with MatP, and the osmolarity of the solutions was adjusted to improve vesicle integrity and yield. Confocal images of the samples and the corresponding intensity profiles showed that green-labeled MatP also bound to the lipid bilayer at the edge of the GUVs (Fig. 2B). Binding to lipids also occurred when MatP was externally added to GUVs (see Fig. S4 in the supplemental material).

10.1128/mBio.00376-19.5 FIG S4 Binding of MatP to the external surface of E. coli lipid vesicles. Shown are representative confocal and transmitted images of GUVs generated from microfluidics droplets containing 150 g/liter Ficoll after external addition of MatP (5 μM) with a trace amount of MatP-Alexa 488. The intensity profile below corresponds to the green channel, obtained across the line drawn in the image. Download FIG S4, TIF file, 0.6 MB. Copyright © 2019 Monterroso et al. 2019 Monterroso et al. This content is distributed under the terms of the Creative Commons Attribution 4.0 International license.

These results showed that the division protein MatP interacts with lipid monolayers or bilayers resembling the composition of the E. coli inner membrane when encapsulated inside micrometer-size cytomimetic containers.

### MatP binds to *E. coli* lipid bilayers at submicromolar concentrations mainly through hydrophobic interactions.

**FIG 3:**
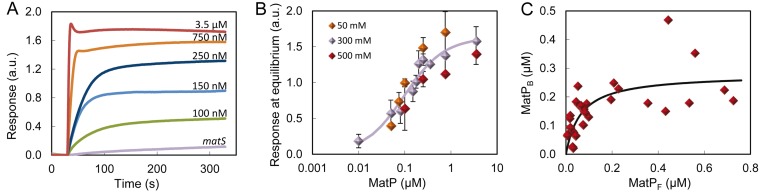
Binding of MatP to E. coli lipids by biolayer interferometry or using lipid-coated microbeads. (A) Representative profiles of the binding of MatP at the indicated concentrations obtained by biolayer interferometry. The profile for *matS* is shown for comparison. (B) Dose-response curves obtained as a function of the concentration of MatP in working buffer with the indicated KCl concentrations. The solid line is the best fit of the model explained in the main text to the data at 300 mM KCl, rendering the following parameter values: *c*_50_ = 0.097 μM and *y*_max_ = 1.6. (C) MatP binding to E. coli lipid-coated microbeads in working buffer plotted as a function of the concentration of free MatP. Symbols represent the data, and the solid line represents the best fit according to the model explained in the main text rendering the parameter values *c*_50_ = 0.065 μM and *y*_max_ = 0.280 μM. The concentration of the beads is 35 g/liter (62 μM accessible lipid). MatP was labeled with Alexa 488.

To quantify the interaction of MatP with lipid membranes, biolayer interferometry assays were conducted using biosensor tips coated with the E. coli lipid mixture. Addition of the protein resulted in a shift in the incident light directed through the biosensor, indicative of binding (Fig. 3A). A dose-response curve obtained by varying the concentration of MatP showed that, above 10 nM, the biosensor signal associated with binding increases with protein concentration, reaching saturation at around 1 μM MatP (Fig. 3B; see Fig. S5 in the supplemental material). Because the precise mode of binding of MatP is not known, and proteins usually interact with lipids in a multivalent manner, an empirical model was fit to this binding curve to obtain an apparent affinity value, as typically done for protein-lipid interactions (23). Analysis with a Langmuir adsorption equation, with no assumption about the mechanism or stoichiometry of the binding, rendered a *c*_50_ value of 0.097 μM, corresponding to the concentration of MatP at which half of the maximum response signal was observed.

10.1128/mBio.00376-19.6 FIG S5 Binding of MatP to lipid bilayers by biolayer interferometry. (A) Dose-response curves of MatP binding to E. coli lipids as a function of protein concentration in the absence (violet) and presence (green) of 1 μM *matS*. Solid lines are the best fit according to the model explained in the main text, rendering the following parameter values: *c*_50_ = 0.097 μM and *y*_max_ = 1.6 for MatP alone and *c*_50_ = 0.076 μM and *y*_max_ = 1.3 for MatP in the presence of *matS*. (B) Probability distribution of *c*_50_ for the binding of MatP to E. coli lipids. Dependence of the relative probability of a given value of *c*_50_ was calculated from a Fisher *F* test for equality of variances. *c*_50_ is in μM. The color code is as in panel A. (C) Dose-response curve of MatP binding to PC as a function of the concentration of MatP. Symbols are data ± standard deviation (SD). The dotted line is only meant to guide the eye. Download FIG S5, TIF file, 0.7 MB. Copyright © 2019 Monterroso et al. 2019 Monterroso et al. This content is distributed under the terms of the Creative Commons Attribution 4.0 International license.

The binding of MatP to lipids was also ascertained through cosedimentation assays using microbeads coated with the E. coli lipid mixture and MatP-Alexa 488. Significant depletion of the protein was observed after incubation with the beads and centrifugation, and the amount of protein bound increased with its concentration at a constant lipid concentration (Fig. 3C; see Fig. S6 in the supplemental material). Observation of the microbeads after incubation with the green-labeled protein by confocal microscopy confirmed the interaction (Fig. S6). The binding isotherm obtained by plotting the concentration of protein bound to the beads against the concentration of free protein was analyzed, as in the biolayer interferometry experiments, using the empirical Langmuir model (Fig. 3C; Fig. S6). This analysis rendered a *c*_50_ of 0.065 μM, close to the midpoint of the response curve obtained by biolayer interferometry. Therefore, these two kinds of binding assays further supported the interaction of MatP with lipids, showing that it occurs at submicromolar concentrations of the protein.

10.1128/mBio.00376-19.7 FIG S6 Binding of MatP to microbeads coated with E. coli lipid bilayers. (A) MatP binding plotted as a function of the total concentration of MatP. Symbols are data ± SD. The concentration of beads was 35 g/liter (62 μM accessible lipid). MatP was labeled with Alexa 488. (B) Representative confocal images of microbeads coated with the E. coli lipid mixture after external addition of MatP-Alexa 488. (C) Probability distribution of *c*_50_ obtained from the best fit of binding data shown in Fig. 3C. Dependence of the relative probability of a given value of *c*_50_ was calculated from a Fisher *F* test for equality of variances. *c*_50_ is in μM. Download FIG S6, TIF file, 0.6 MB. Copyright © 2019 Monterroso et al. 2019 Monterroso et al. This content is distributed under the terms of the Creative Commons Attribution 4.0 International license.

To determine the nature of the interactions of MatP with lipids, the biolayer interferometry assays were repeated at lower and higher KCl concentrations: 50 and 500 mM (Fig. 3B). No significant difference in the binding profile was observed at either salt concentration with respect to that obtained in working buffer (300 mM KCl), except for a slight shift toward lower concentration values at 50 mM KCl. These results were further confirmed using lipid-coated microbeads showing MatP-bound fractions of 0.879 ± 0.003, 0.79 ± 0.06, and 0.79 ± 0.05 at 50, 300, and 500 mM KCl, respectively. This relatively insensitive response to changes in ionic strength rules out a major contribution of electrostatic interactions in the recognition of lipids by MatP. The fact that MatP also binds to bilayers of phosphatidylcholine (PC), a neutral lipid, further supports this conclusion, pointing toward hydrophobic interactions as the most likely driving force of lipid recognition by this protein (Fig. S5C).

### MatP does not recruit *matS* to the membrane.

As MatP is a DNA binding protein, we asked if it was still able to bind to the E. coli lipids in the presence of oligonucleotides containing its specific binding sequence, *matS*. To approach this question, we first characterized the protein/DNA complexes in the working buffer used to study MatP binding to lipids. MatP behaved as a dimer, according to sedimentation and light scattering data (see analysis of MatP/*matS* complexes in Fig. S7 and the associated supplemental material), in good agreement with previous reports (24). The stoichiometry of the MatP/*matS* complex was found to be two monomers of MatP and one molecule of the *matS* target (Fig. S7), again in agreement with previous analysis (24). The *K_d_* (dissociation constant) for the interaction, determined by fluorescence anisotropy using fluorescein-labeled *matS* (*matS*-Fl), was 15 ± 2 nM in dimer units (see analysis of MatP/*matS* complexes in Fig. S7 and the associated supplemental material).

10.1128/mBio.00376-19.8 FIG S7 Characterization of MatP/*matS* complexes in solution by fluorescence anisotropy, analytical ultracentrifugation, and light scattering. Also included are a description of results and associated materials and methods. Download FIG S7, PDF file, 0.5 MB. Copyright © 2019 Monterroso et al. 2019 Monterroso et al. This content is distributed under the terms of the Creative Commons Attribution 4.0 International license.

**FIG 4:**
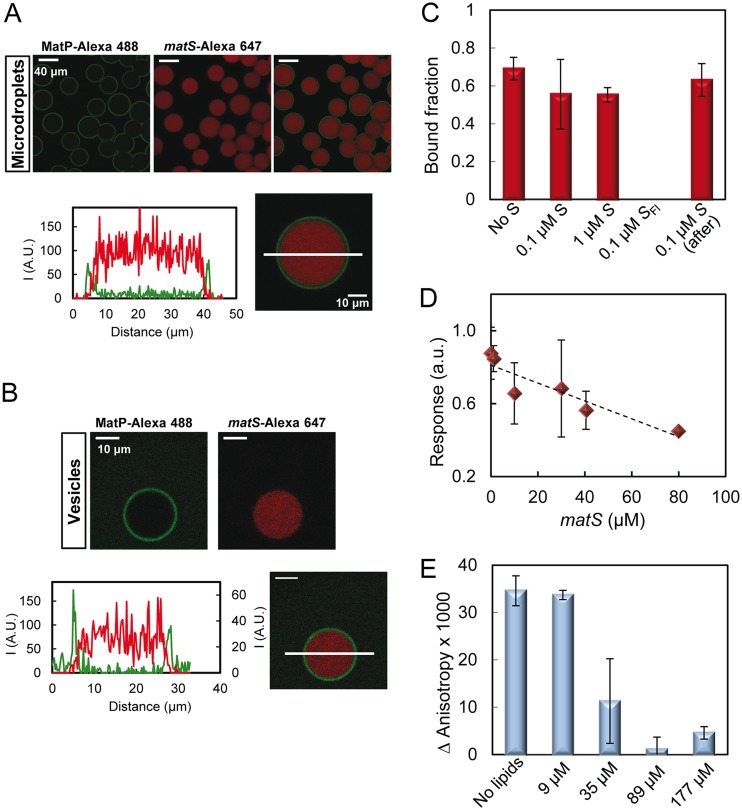
Binding of MatP to lipids in the presence of *matS.* (A and B) Representative confocal images of microdroplets and GUVs, respectively, stabilized by the E. coli lipid mixture containing MatP and *matS*, and intensity profiles below. Profiles correspond to the green (MatP-Alexa 488 [1 μM]) and red (*matS*-Alexa 647 [1 μM]) channels, obtained across the line as drawn in the images. The concentrations of MatP and *matS* were 3.5 and 1.4 μM, respectively. Vesicles also contained 150 g/liter Ficoll. (C) Effect of *matS* on MatP binding to lipid-coated beads (30 g/liter, 53 μM accessible lipid). The MatP concentration in all measurements was 0.25 μM. Unless otherwise stated, the labeled species was MatP-Alexa 488. *S* and *S*_Fl_ represent *matS* and *matS* labeled with fluorescein, respectively. For the measurement corresponding to the bar on the far right, *matS* was added to MatP already bound to the lipid. (D) Competition of *matS* with the lipids for binding to MatP as observed by biolayer interferometry. The concentration of MatP was 0.15 μM. The dashed line is only meant to guide the eye. (E) Fluorescence anisotropy-based competition between lipids and DNA for MatP. Shown is the anisotropy change of *matS*-Fl (50 nM) in the presence of MatP (100 nM) as a function of the concentration of accessible lipid coating microbeads.

We next encapsulated MatP along with *matS* inside cell-like containers to analyze the influence of the oligonucleotide on MatP interaction with the lipids. Encapsulation of MatP (with a trace amount of MatP-Alexa 488) and *matS*-Alexa 647 showed that the location of MatP, almost exclusively at the lipid boundary of the microdroplets or GUVs, was not altered by the presence of *matS*, while the DNA, in turn, remained homogeneously distributed in their lumen (Fig. 4A and B). Remarkably, the intensity profiles showed a drop of the *matS* red signal at the edges of the vesicle, where the green signal corresponding to MatP reaches its maximum. This strongly suggests that MatP at the membrane is not bound to the DNA. The concentrations of MatP and *matS* in these experiments were well above their *K_d_* of interaction, and we used a protein (monomer) molar excess relative to the DNA concentration above 2-fold to ensure formation of the 2:1 complex previously characterized in solution (described above). The same results were found either by including MatP and *matS* in two independent streams, triggering complex formation shortly before encapsulation, or by encapsulating the preformed complex (i.e., MatP and *matS* together in the two streams). Additional experiments in which the fluorescein-labeled *matS* used in the fluorescence anisotropy binding titrations and unlabeled MatP were encapsulated showed, again, that the DNA remained in the lumen of the microdroplets (see Fig. S8A in the supplemental material). The images obtained in this case were indistinguishable from those corresponding to the encapsulation of fluorescein-labeled *matS* alone (Fig. S8B). These experiments evidenced that MatP still binds to the lipid monolayers or bilayers of microdroplets and GUVs in the presence of *matS*, although there was no sign of concomitant DNA recruitment to the lipid edge.

10.1128/mBio.00376-19.9 FIG S8 Representative confocal and transmitted images of microdroplets generated by manual bulk emulsion using different combinations of labeled MatP and *matS*. (A) MatP and *matS* with either labeled *matS* (2.5 μM MatP and 1 μM *matS* [top]) or labeled MatP (1.7 μM MatP and 0.8 μM *matS* [bottom]). (B) *matS* (1.4 μM [top]) and MatP (1.7 μM [bottom]). Intensity profiles on the right correspond to the green channel, obtained across the line drawn in the images. Download FIG S8, TIF file, 2.7 MB. Copyright © 2019 Monterroso et al. 2019 Monterroso et al. This content is distributed under the terms of the Creative Commons Attribution 4.0 International license.

Next, we probed the influence of *matS* on the binding of MatP to lipids using microbeads coated with the E. coli lipid mixture and through biolayer interferometry. Addition of 0.1 to 1 μM unlabeled *matS* prior to or after incubation of MatP with the microbeads did not significantly modify the fraction of MatP-Alexa 488 bound with respect to that in the absence of *matS* (Fig. 4C). Parallel experiments using fluorescein-labeled *matS* and unlabeled MatP showed that the DNA did not bind to the lipids together with MatP (Fig. 4C), in good agreement with the images of the complex encapsulated inside lipid vesicles or microdroplets.

Biolayer interferometry assays conducted to measure the binding of MatP in the presence of a constant 1 μM concentration of *matS* rendered isotherms of binding superimposable, within error, with those obtained in the absence of *matS* (Fig. S5; *c*_50_ = 0.076 μM), and no significant interaction with the lipids was detected for *matS* alone (Fig. 3A). The signal of binding of MatP (150 nM) to the lipids showed a decreasing trend with *matS* concentration (Fig. 4D), more obvious at high concentration, which suggests competition between the lipids and the DNA for binding to the protein. No such effect was observed under conditions under which the interaction between MatP and *matS* is greatly hindered while still allowing significant binding of MatP to the lipids (500 mM KCl, 150 nM MatP [see Fig. S9 in the supplemental material]), implying the alleged competition observed at lower salt concentrations would arise from the interaction between MatP and *matS*.

10.1128/mBio.00376-19.10 FIG S9 Effect of *matS* on the binding of MatP to lipids at different salt concentrations, monitored by biolayer interferometry. The concentration of MatP was 0.15 μM. The inset shows fluorescence anisotropy binding titrations of *matS*-Fl (10 nM) with MatP in buffer with 500 mM KCl. Symbols are the average from 3 individual replicates ± SD at the specified salt concentrations. Solid lines are only intended to guide the eye. Download FIG S9, TIF file, 0.4 MB. Copyright © 2019 Monterroso et al. 2019 Monterroso et al. This content is distributed under the terms of the Creative Commons Attribution 4.0 International license.

Competition between *matS* and the membrane for binding to MatP was also observed by fluorescence anisotropy. The formation of complexes of fluorescein-labeled *matS* with MatP led to an increase in the anisotropy with respect to that of free *matS*-Fl (Fig. 4E). Incubation of MatP/*matS*-Fl complexes with lipid-coated microbeads and subsequent centrifugation to sediment the beads resulted in a concentration-dependent decrease in the anisotropy value compared to that in the absence of lipids (Fig. 4E). At sufficiently high lipid concentration, anisotropy reached values close to that of the free *matS*, compatible with total dissociation of the protein from the DNA because of lipid competition. The fluorescence intensity remained unchanged across the titration, as expected for competition between the lipids and *matS*, rather than recruitment of the latter to the lipids concomitantly with the protein.

Further interpretation of these competition experiments and of the results obtained with encapsulated MatP/*matS* complex (see above) by direct comparison of the affinity values determined is not straightforward. While a thermodynamic binding constant was attained for the MatP/*matS* complex through precise determinations of its stoichiometry, only apparent affinity constants could be determined for the interaction of MatP with lipids, given the unknown stoichiometry in this case. Besides, the self-association of the protein should also be taken into account in the interaction scheme, and the amount of MatP bound to *matS* or to the membrane in each situation would depend both on the relative affinities of the protein for the two ligands and on their concentrations. For example, encapsulated MatP finds a concentration of *matS* that is probably too low to reach competition in the presence of an excess of lipid, like that within the boundary, which may explain why the protein is shifted toward the lipid surface despite the presence of *matS*.

Taken together, these experiments show that *matS* and the lipids compete for binding to MatP instead of forming ternary complexes, not necessarily implying overlap between the lipid and nucleic acid binding regions of the protein.

## DISCUSSION

Here we have found that the protein of the Ter linkage MatP interacts with membranes matching the lipid composition of the E. coli inner membrane, as shown by encapsulation in cell-like containers, cosedimentation with lipid-coated microbeads, and biolayer interferometry assays. Although MatP presents dual recognition of lipids and nucleic acid sequences, we have not found any indication supporting the formation of ternary complexes, strongly suggesting that both types of ligands may be mutually exclusive, which is also illustrated by the predominantly axial localization of the MatP foci.

The interaction of MatP with the membrane seems to be driven by hydrophobic rather than electrostatic forces, given the minor impact of salt variations and the tendency of the protein to recognize neutral lipids. An interaction of an electrostatic nature could have been expected, as the E. coli lipid mixture contains negatively charged phospholipids (phosphatidylglycerol and cardiolipin [25]), while given its high pI, MatP would be positively charged at neutral pH. However, our experiments rule out a major contribution of this kind of interactions, suggesting in turn that the recognition could be the result of hydrophobic protein-lipid contacts. A domain of MatP that may be involved in this type of binding is the C-terminal coiled coil, previously suggested to be responsible for tetramerization (15). This domain is away from the N-terminal modules (the four-helix bundle and the RHH) specifically targeting the *matS* sequences. Lipid binding through the coiled-coil domain or any other region of MatP outside the DNA binding domains would not be incompatible with the lack of DNA recruitment to the membrane. Indeed, overlap between the lipids and DNA binding sites is only one of the possible explanations for this finding—not even the most probable one—in view of the mainly hydrophobic interaction with lipids and the charged nature of the DNA binding modules. Other possible scenarios leading to the same experimental observation would involve lipid-induced changes in the association state, orientation, and/or conformation of MatP hampering DNA binding or some sort of steric hindrance between the two ligands, precluding their simultaneous recognition. Further studies will be required to determine the precise elements within MatP structure responsible for the interaction with lipids.

**FIG 5:**
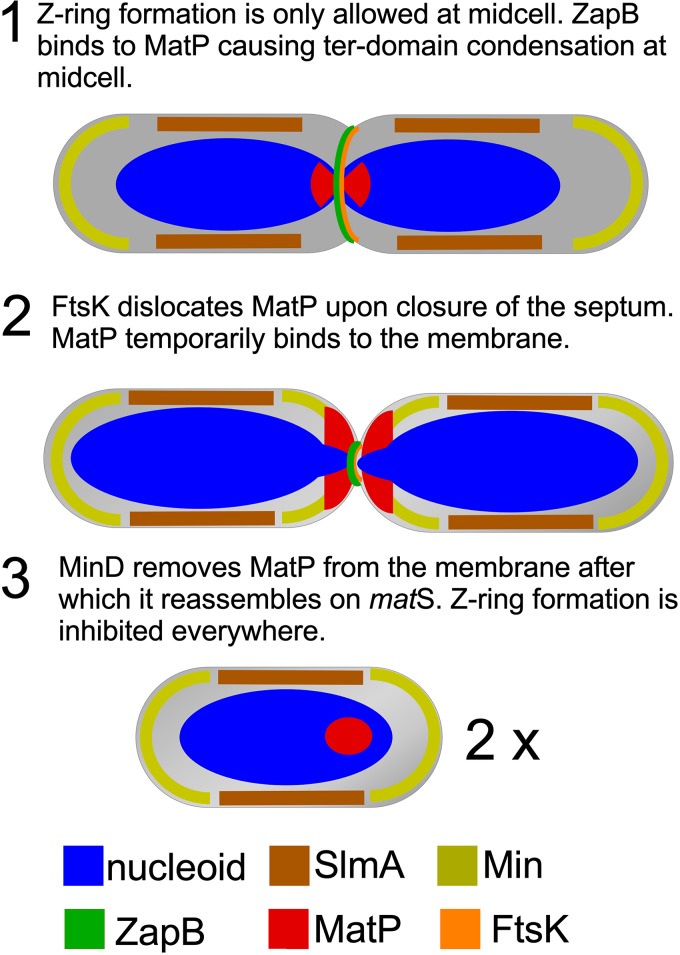
Hypothetical model of the MatP dissociation from the *matS* sites and its binding to the cytoplasmic membrane. In step 1, ZapB is binding MatP at midcell, causing the 23 *matS* sites to cluster and ensuring that the terminus remains at midcell. Z-ring formation is inhibited at the old poles by the Min system and in the cylindrical part of the cell close to the bulk of the nucleoid, but not in the Ter domain, by the nucleoid occlusion protein SlmA. The brown bar indicates the nucleoid occlusion by SlmA, not the binding of SlmA to the nucleoid. In step 2, the nucleoids are segregating and MatP is pulled away from ZapB. At the same time, the terminus is bound by FtsK that displaces MatP from *matS* sites by translocation of the DNA near the terminus, which allows final segregation of the nucleoids into the daughter cells. In step 3, MinD removes MatP from the membrane, after which it reassembles on *matS*. Z-ring formation is inhibited everywhere by the Min system and the nucleoid occlusion protein SlmA.

Membrane binding of MatP may serve to sequester the protein from the chromosome under conditions in which its positive regulation of Z-ring formation is no longer required and even might obstruct the function of proteins like FtsK. FtsK is needed for the deconcatenation of sister chromosomes and helps to segregate the termini into each daughter cell (9, 26). FtsK, part of the divisome (27), is one of the fastest DNA translocases (28). Membrane binding of MatP released by FtsK during this relatively short time interval might function to prevent rebinding to the *matS* sites close to the *dif* site. We propose that competition between *matS* and lipid for MatP assists in the segregation of the *dif* region by FtsK during the last step of septum closure (Fig. 5). Then, MatP would be subsequently released from the membrane to bind again the *matS* sites. A possibility is that, as part of the oscillation of the Min system between the old poles and the newly formed septum before daughter cells have separated (29), detachment from the membrane might be assisted by MinD, known to displace proteins from the membrane surface of the new poles (30, 31). Testing this hypothesis may be the subject of future research on this system.

Recent studies have revealed that, like MatP, other proteins binding to the bacterial chromosome are also able to interact with lipid membranes. Examples of these proteins are the nucleoid occlusion protein Noc from Bacillus subtilis (18), a negative modulator of Z-ring assembly, SeqA from E. coli, a protein involved in the sequestration of replication origins (32), the proline utilization flavoprotein PutA (33), and the SOS repair system regulator RecA (34). Along the same line, the nucleoprotein complexes of SlmA, the factor counteracting Z-ring formation around the chromosome in E. coli, seem to be brought close to the membrane (4, 35, 36), possibly through transertional linkages (36) and/or biomolecular condensation (37). Conversely, well-known membrane-associated proteins like MinD from the Min system (38) have also been shown to interact, in a non-sequence-specific manner, with chromosomal DNA (39).

The main difference between the dual recognition of lipids and DNA by MatP and the other site selection proteins (Noc [18] and MinD [38]) is that the latter can simultaneously bind DNA and lipids. Furthermore, in the particular case of Noc, binding to DNA activates the subsequent interaction with the membrane (18). The precise mode of recognition may serve the function of each regulator and may be related to the structural elements involved in membrane binding—amphipathic helixes in the case of MinD (38) and Noc (18)—which we have not found in MatP by using AMPhipaseek software (40). In bacillary bacteria, DNA-membrane interactions are proposed to aid in the localization of the bacterial cell center, where the strength of these interactions decreases and Z-ring assembly is favored (41). According to this, by bridging the chromosome and the membrane, negative regulators of division ring formation such as Noc would exclude FtsZ from noncentral areas, biasing FtsZ assembly to the midcell (18). In contrast, physical connection of the chromosome with the membrane through MatP could interfere with its positive regulation of FtsZ assembly that contributes to division ring positioning. In the last step of binary fission that requires deconcatenation of sister chromosomes and closure of the septum, the presence of MatP might not be beneficial any longer. Therefore, it may be displaced to the membrane to prevent immediate rebinding to the Ter domain, which would happen otherwise given its high affinity for these sites.

Other E. coli proteins have been reported to interact with membrane and DNA in a mutually exclusive way, as seems to be the case of MatP. One of these proteins is PutA, which interacts with the membrane primarily through hydrophobic contacts (33) and contains a DNA binding RHH domain involved in dimerization (42), similarly to MatP (15). To explain the incompatibility of simultaneous DNA and membrane binding, the formation of multimeric complexes has been suggested, implying the membrane binding domain could be constrained in these complexes and, likewise, membrane-bound protein would constrain the DNA binding domain, hindering its recognition (42). In the case of the protein RecA, the domains for the interaction with DNA and lipids are located on different sides of the protein, which upon membrane interaction, forms polymeric structures, preventing its association with the chromosome (43). In both cases, membrane binding in the presence of ligands is reversible, implying protein-membrane associations are in equilibrium with the protein/DNA complex, which was also observed in the MatP competition experiments we report here. It remains to be determined whether the binding partners of MatP may also tune its interaction with both DNA and membrane.

Since its identification, the function of MatP and its modulation in the context of division have been traditionally linked to its specific binding to DNA sequences within the Ter macrodomain or to its interaction with other proteins such as ZapB. Our findings strongly suggest that, in addition to protein-nucleic acid and protein-protein interactions, protein-lipid recognition should also be taken into account in the analysis of the function of MatP. Further work will be required to elucidate the precise mechanisms of these protein-membrane interactions and the factors influencing them.

## MATERIALS AND METHODS

### Chemicals and reagents.

Polar extract of E. coli phospholipids, from Avanti Polar Lipids (Alabaster, AL), was stored in chloroform at −20°C. Analytical-grade chemicals were from Sigma. Silica microbeads were from Bangs Laboratories. Alexa Fluor 488 carboxylic acid succinimidyl ester dye was from Molecular Probes-Thermo Fisher Scientific. High-performance liquid chromatography (HPLC)-purified oligonucleotides containing the *matS19* sequence targeted by MatP (AAA**GTGACACTGTCAC**CTT [bases recognized by the protein are in boldface]) (5), with or without fluorescein or Alexa 647 covalently attached to the 5′ end of the sense oligonucleotide, were purchased from Microsynth or IDT. Complementary strands were hybridized by heating at 85°C in a thermocycler and slowly cooling down. The fluorescently labeled oligonucleotides (*matS*-Fl or *matS*-Alexa 647) were hybridized with a 10% excess of the unlabeled complementary strand. Unless otherwise stated, *in vitro* experiments were done in 50 mM Tris-HCl, 300 mM KCl, and 5 mM MgCl_2_ at pH 7.5 (working buffer).

### Bacterial strains and growth conditions.

MG1655 *matP-mCh*::*kan*, a kind gift of Pauline Dupaigne (15), was grown to steady state at 28°C while shaking at 205 rpm in minimal glucose medium (Gb4 medium): 6.33 g K_2_HPO_4_ (Merck), 2.95 g KH_2_PO_4_ (Riedel de Haen), 1.05 g (NH_4_)_2_SO_4_ (Sigma), 0.10 g MgSO_4_⋅7H_2_O (Roth), 0.28 mg FeSO_4_⋅7H_2_O (Sigma), 7.1 mg Ca(NO_3_)_2_⋅4H_2_O (Sigma), 4 mg thiamine (Sigma), 50 mg lysine (Sigma), 50 mg arginine (Sigma), 50 mg glutamine (Sigma), 2 mg thymidine (Sigma), 20 mg/liter uracil (Sigma) and 4 g glucose per liter at pH 7.0. At an optical density at 450 nm (OD_450_) of 0.2 (Biochrom Libra S70 spectrophotometer; Harvard Biosciences), cells were fixed by 2.8% formaldehyde and 0.04% glutaraldehyde for 15 min before being washed in phosphate-buffered saline (PBS) (44). After being split into two batches, one batch was immunolabeled with antibodies against ZapB and the other with antibodies against FtsN (14) as described previously (44). The nucleoids were then stained with 1 μg/ml DAPI. Secondary antibodies were donkey anti-rabbit IgG conjugated to Oregon Green (Jackson Immunoresearch). When cells were imaged live, they were concentrated and resuspended gently in their own medium. Expression of pXL28 mNG-GlpT was induced for 2 mass doublings with 15 or 30 μM IPTG (isopropyl-β-d-1-thiogalactopyranoside [Duchefa]) for wide-field fluorescence microscopy and structured illumination microscopy, respectively.

### Microscopy and image analysis.

For imaging, the cells were immobilized on 1% agarose in water slabs on object glasses (45), and phase-contrast and fluorescence microscopy images were obtained using a Nikon Eclipse Ti microscope equipped with a C11440-22CU Hamamatsu ORCA camera, an Intensilight HG 130-W lamp, and NIS Elements software (version 4.20.01). Images were analyzed with Coli-Inspector supported by the ObjectJ plugin for ImageJ (version 1.49v) (14). Calculation of the Pearson coefficient of the colocalization of MatP and GlpT was determined as described previously (20).

### SIM sample preparation and imaging.

Micrometer holes (1.1 to 1.4 μm) were made with a micropillar mold in a 3% agarose in a Gb4 medium layer to orient the cells vertically (46). To immobilize the cells in these holes, a thin layer of 1% low-melting-point agarose in Gb4 medium was applied on top. A cover glass was then applied and taped to the glass slide. Imaging settings are described in the supplemental material (Text S1).

10.1128/mBio.00376-19.1 TEXT S1 *In vivo* imaging. Information about additional materials and methods is provided. Download Text S1, PDF file, 0.1 MB. Copyright © 2019 Monterroso et al. 2019 Monterroso et al. This content is distributed under the terms of the Creative Commons Attribution 4.0 International license.

### MatP expression, purification, and labeling.

Recombinant untagged MatP was produced as previously described (15), with some modifications, from the plasmid kindly provided by M Schumacher. Briefly, the N-terminal hexahistidine (His_6_)-tagged protein was overproduced and purified by affinity chromatography using a His-bind resin (Novagen) with nickel. The His_6_ tag was subsequently removed by cleavage with thrombin, followed by an ion-exchange chromatography step using a HiTrap SP HP column (GE Healthcare). The fractions of MatP were pooled, dialyzed against 50 mM Tris-HCl, 300 mM KCl, 1 mM EDTA, and 10% glycerol at pH 7.5, and stored at −80°C. The protein concentration was measured by UV-absorbance spectroscopy using a molar absorption coefficient at 280 nm of 27,960 M^−1^cm^−1^, estimated from its sequence. MatP was covalently labeled in the amino groups with Alexa Fluor 488 carboxylic acid succinimidyl ester dye (MatP-Alexa 488) at pH 7.5 to favor selective labeling of the N-terminal residue of the protein (47) and stored at −80°C. The ratio of labeling was around 0.5 mol of fluorophore per mol of protein, as estimated from their molar absorption coefficients. The interaction of the labeled protein with *matS* was confirmed through sedimentation velocity experiments (Fig. S7C).

### Microfluidic encapsulation in microdroplets, generation of giant unilamellar vesicles, and visualization by confocal fluorescence microscopy.

Microfluidic devices were constructed by conventional soft lithographic techniques from masters (chip design and procedure detailed elsewhere [48]). Encapsulation was conducted at room temperature by mixing in a 1:1 ratio, prior to the droplet formation junction, the stream of MatP solution with that of buffer including (when stated) *matS*-Alexa 647. When present, both aqueous streams contained crowders (Ficoll or dextran). The third stream supplied the E. coli lipid mixture at 20 to 25 g/liter in mineral oil, prepared shortly before use by two cycles of vortex/sonication and resuspension in the mineral oil of a lipid film obtained using a SpeedVac device. Encapsulation was also conducted including the preformed MatP/*matS* complex in the two aqueous streams. The data presented correspond to experiments delivering solutions at 160 and 20 μl/h (oil and aqueous phases, respectively) by automated syringe pumps (Cetoni GmbH), yielding uniform droplets that were collected for 30 min for their subsequent conversion into giant unilamellar vesicles following the procedure described elsewhere (21).

Microfluidic production of droplets on the chip was monitored with an Axiovert 135 fluorescence microscope (Zeiss). The resulting microdroplets and GUVs were visualized immediately after generation by confocal microscopy with Leica TCS-SP2 or TCS-SP5 inverted confocal microscopes as previously described (21, 49). Intensity profiles in the green and red channels were obtained applying the line tool of ImageJ (National Institutes of Health) through the equatorial section of the droplets/vesicles.

### Biolayer interferometry measurements.

Lipid-protein interactions were measured by biolayer interferometry using a single-channel BLItz system (ForteBio). Lipids were immobilized on aminopropylsilane biosensor tips by immersion into a 0.5 g/liter small unilamellar vesicle (SUV) solution freshly prepared before the experiments following a procedure earlier described (50). MatP binding to the immobilized lipids, with or without *matS*, was measured at the specified final protein concentrations at room temperature and with vigorous shaking (2,200 rpm). Assays were performed at least by duplicate, and binding isotherms were constructed by representing the experimental binding values at equilibrium versus the MatP total concentration.

### Binding assays in lipid-coated microbeads.

Microbead coating, binding measurements, and the calculation of the amount of lipid coating the microbeads were done as described previously (51). Binding experiments at different salt concentrations (50, 300, and 500 mM KCl) were conducted using MatP-Alexa 488 (125 nM Alexa 488, 250 nM MatP) and 20 g/liter beads (35 μM accessible lipids). Experiments in the presence of *matS* were performed by adding, prior to or after incubation with the attached lipids, unlabeled *matS* (0.1 or 1 μM) to the samples containing MatP-Alexa 488 (0.250 μM). Additionally, *matS*-Fl (0.1 μM) was added to samples containing unlabeled MatP (0.250 μM) and lipids. After 20 min of incubation of the protein or the nucleoprotein complex with the coated beads, samples were centrifuged, and free protein/nucleoprotein complex remaining in the supernatant was quantified using a fluorescence plate reader (Varioskan Flash, Thermo Scientific, or POLARstar Galaxy, BMG Labtech) as described previously (51). Assays were performed in triplicate, and the binding isotherm was constructed by plotting the concentration of bound MatP as a function of the concentration of free MatP. The linearity of the signal of the labeled protein with its concentration was verified.

### Anisotropy-based competition between lipids and DNA for MatP.

Fluorescence anisotropy measurements were performed on the MatP/*matS* complexes (100 nM MatP, 50 nM *matS*-Fl), in the presence of increasing amounts of lipid-coated microbeads. The three elements (MatP, *matS*, and the microbeads) were incubated and centrifuged at low speed to sediment the beads (51), and the anisotropy of the material remaining in the supernatant was measured (see the supplemental information associated with Fig. S7 for details on the anisotropy measurements).

### Analysis of protein-lipid binding isotherms.

Binding parameters were obtained independently from the analysis of the isotherms from interferometry or microbead assays by a nonlinear least-squares fit of a Langmuir adsorption isotherm

$$y=y_{\max}\frac{\left(c/c_{50}\right)}{1+\left(c/c_{50}\right)}$$

where *y* and *y*_max_ are the response and maximum response measured upon binding, respectively, *c* is the concentration of MatP, and *c*_50_ is the concentration of MatP at which binding is half of the maximum value.

The method of parameter scanning (52) was employed to determine the extent to which the value of the best-fit parameter is determined by the data, as explained elsewhere (51).
