# Supplementary material for: The Bacterial DNA Binding Protein MatP Involved in Linking the Nucleoid Terminal Domain to the Divisome at Midcell Interacts with Lipid Membranes
Source: mBio. 2019 May 28;10(3):e00376-19. doi: 10.1128/mBio.00376-19 (PMC6538776; doi:10.1128/mBio.00376-19)
Supplement: FIG S7 [file mBio.00376-19-sf007.pdf]

Analysis of MatP·*matS* complexes

1. Results

Fluorescence anisotropy binding titrations showed that, in working buffer (300 mM KCl), purified MatP binds to a short fluorescein labelled double stranded oligonucleotide containing the *matS19* sequence (1) with high affinity in the nanomolar concentration range (**Fig. S7A**). To propose suitable models for the analysis of the binding isotherm, the stoichiometry of the MatP·*matS* complex and the oligomerization state of MatP were studied by sedimentation velocity and SEC-MALLS (**Fig. S7B**). Both *matS* and MatP sedimented as single species with 2.3S and 2.1S, respectively, compatible with the 19-mer double stranded DNA and a dimer of the protein, respectively, as confirmed by light scattering (inset in **Fig. S7B**). The association state of MatP was insensitive to its concentration in the 0.5-9 μM range (2.3S and 2.0S, respectively). When both MatP and *matS* were present, sedimentation velocity profiles showed the appearance of a 3.5S peak, corresponding to the nucleoprotein complex, that increased from 52% to 89% with increasing concentrations of MatP at the expense of the free DNA species at ~2S. SEC-MALLS experiments indicated that this complex contained two monomers of MatP and one molecule of the *matS19* target. Interestingly, no intermediate or higher order complexes were observed when 1.5:1 or 5:1 protein (monomer): DNA ratios were studied. Analysis of the anisotropy binding isotherm assuming, hence, a model in which one dimer of the protein binds to the DNA forming a single complex, according to the stoichiometries obtained by SV and SEC-MALLS, rendered a K_d for the interaction of 15 ± 2 nM, in dimer units (ΔG = 10.7 ± 0.1 kcal/mol, for the dissociation reaction).

Fluorescence anisotropy measurements of the binding of MatP to *matS*-Fl in buffer with 500 mM KCl showed (see inset in **Fig. S9**) a substantial decrease in the affinity of the interaction.

2. Methods

Fluorescence anisotropy binding measurements. Anisotropy experiments of the binding of MatP and *matS*-Fl in working buffer or in buffer with 500 mM KCl were performed in a PolarStar Galaxy Plate Reader (BMG Labtech) at 26°C, with 485 and 520 nm excitation and emission filters, respectively. Reported anisotropy values are the average of 3-5 independent experiments, each resulting from the average of three measurements obtained after reaching equilibration. The concentration of fluorescein labelled *matS* in the titrations with MatP was 10 nM. This concentration was selected as a compromise to determine binding parameters under equilibrium conditions while still getting a good signal to noise ratio. The emission intensity of fluorescein remained unchanged in the presence of MatP. The binding isotherm determined in working buffer was analysed with BIOEQS software (2) using the model described above, in terms of the free energy of formation of the postulated complex from its individual components. Associated errors were assessed by rigorous confidence

limit testing at the 67% with the same software. BIOEQS uses a numerical solver engine based on a Marquardt-Levenberg algorithm, allowing analysis of binding isotherms with no assumption regarding the concentration of any of the species involved in the interaction (2, 3). This is important as, due to instrumental limitations, the concentration of DNA used in the titrations was only slightly lower than the K_d retrieved for the interaction, a condition that would have precluded the use, if needed, of simplified analysis models that assume the concentration of free protein equals that of total protein across the titration (4-6).

Sedimentation velocity experiments. Sedimentation velocity experiments were carried out at 48,000 rpm and 20°C in an XL-I analytical ultracentrifuge (Beckman-Coulter Inc.) equipped with both UV-VIS and Raleigh interference detection systems, using an An-50Ti rotor and 12 mm double sector centrepieces. Concentrations of *matS* and MatP, in working buffer, are specified in the legend of the figure. In the samples aimed at determining the stoichiometry of the MatP·*matS* complex, different concentration ratios were tested, as specified in the figure. Control experiments were also conducted using unlabelled *matS* and Alexa 488 labelled MatP (**Fig. S7C**). All sedimentation profiles were recorded at 260 nm. The sedimentation coefficient distributions were calculated by least squares boundary modelling of sedimentation velocity data using the *c(s)* method as implemented in SEDFIT (7), and the calculated *s* values corrected to standard conditions (water, 20°C, and infinite dilution) using SEDNTERP (8).

Size exclusion chromatography coupled to multiangle static light scattering. MALLS measurements were conducted in a DAWN-EOS multiangle light scattering photometer (Wyatt Technology Corp) equipped with an Optilab rEX differential refractometer configured to collect data in parallel from the sample eluting from a coupled dextran-agarose column (Superdex 200 10/300 GL, Pharmacia Biotech, flow rate 0.5 mL/min) at 20°C in working buffer. Loading solutions of MatP (16 μM) and MatP·*matS* (16 and 4 μM) were freshly prepared as detailed elsewhere (9). The acquired raw data consisted of the scattering intensity at fourteen scattering angles and the differential refractive index of the species fractionated in the column. Data were acquired and analysed using ASTRA (V.4.90, Wyatt Technology), calculating the average molecular masses from the ratio of scattering to concentration at the peak maximum.

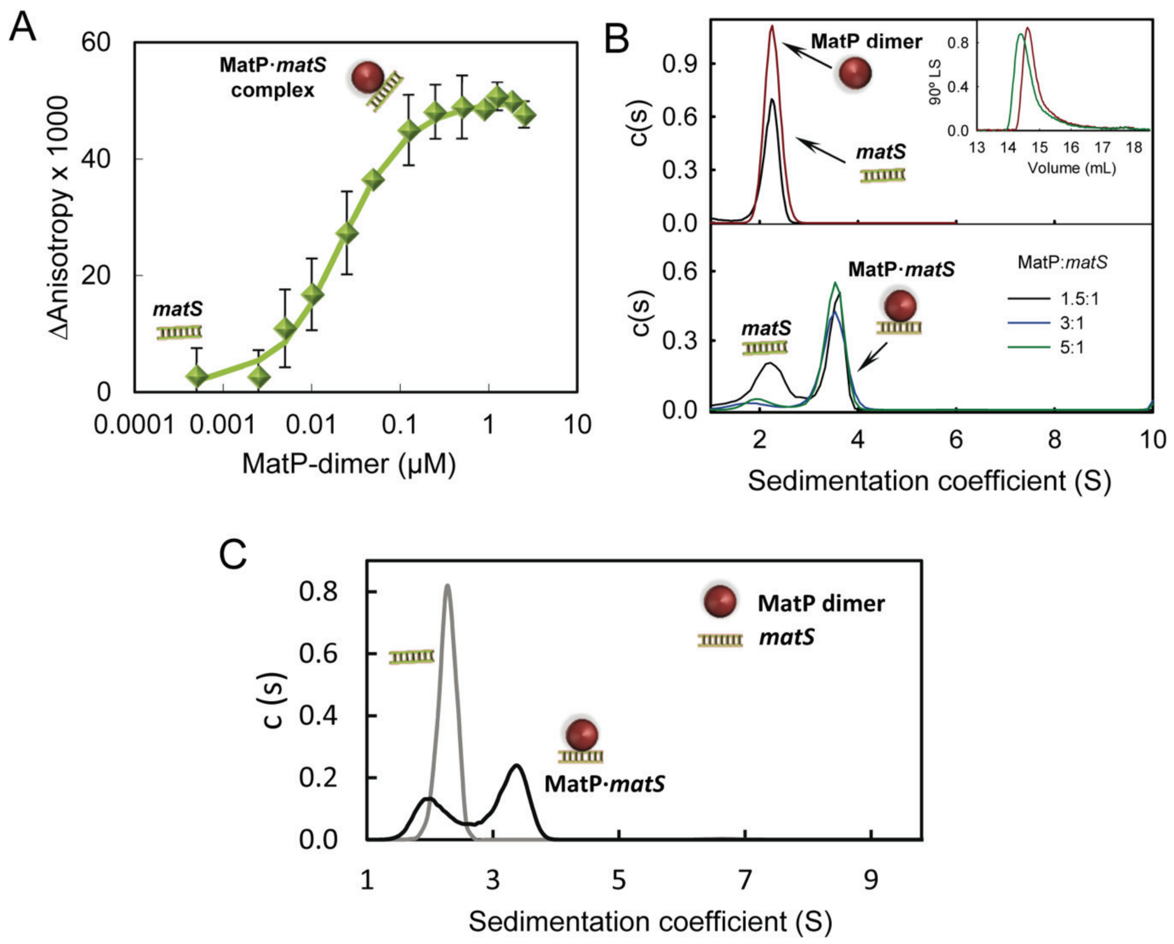

Figure S7. Characterization of MatP·*matS* complexes in solution. (A) Fluorescence anisotropy binding titrations of fluorescein labelled *matS* (10 nM) with MatP. Symbols are the average of five individual replicates ± SD. Solid line indicates the fit of the specified model. (B) SV profiles of MatP (3 μM) and *matS* (0.6 μM) and, below, of the nucleoprotein complex at the specified ratios. Inset, SEC-MALLS profiles of MatP without (red) and with *matS* (green). Determined masses were (35 ± 1) 10^3 and (46 ± 1) 10^3, respectively. (C) Sedimentation profile of MatP·*matS* complexes with labelled MatP. In black, SV profile of MatP-Alexa 488 (1.8 μM) and *matS* (0.6 μM). Profile of DNA at the same concentration is shown for comparison (grey).

Supplementary references

1. Mercier R, Petit MA, Schbath S, Robin S, El Karoui M, Boccard F, Espeli O. 2008. The MatP/matS site-specific system organizes the terminus region of the E. coli chromosome into a macrodomain. Cell 135:475-85.

2. Royer CA, Smith WR, Beechem JM. 1990. Analysis of binding in macromolecular complexes: a generalized numerical approach. Anal Biochem 191:287-94.
3. Royer CA, Beechem JM. 1992. Numerical analysis of binding data: advantages, practical aspects, and implications. Methods Enzymol 210:481-505.
4. Beckett D. 2011. Measurement and analysis of equilibrium binding titrations: A beginner's guide. Methods Enzymol 488:1-16.
5. Pollard TD. 2010. A guide to simple and informative binding assays. Mol Biol Cell 21:4061-7.
6. Rippe K. 1997. Analysis of protein-DNA binding at equilibrium. BIF Futura 12:20-26.
7. Schuck P. 2000. Size-distribution analysis of macromolecules by sedimentation velocity ultracentrifugation and lamm equation modeling. Biophys J 78:1606-19.
8. Laue TM, Shah, B.D., Ridgeway, T.M., Pelletier, S.L. . 1992. Interpretation of analytical sedimentation data for proteins In: Harding SE, Rowe AJ, Horton JC, editors. Analytical ultracentrifugation in biochemistry and polymer science. , Cambridge, UK: Royal Society of Chemistry.
9. Cabre EJ, Monterroso B, Alfonso C, Sanchez-Gorostiaga A, Reija B, Jimenez M, Vicente M, Zorrilla S, Rivas G. 2015. The Nucleoid Occlusion SlmA Protein Accelerates the Disassembly of the FtsZ Protein Polymers without Affecting Their GTPase Activity. PLoS One 10:e0126434.
