# Supplementary material for: The Bacterial DNA Binding Protein MatP Involved in Linking the Nucleoid Terminal Domain to the Divisome at Midcell Interacts with Lipid Membranes
Source: mBio. 2019 May 28;10(3):e00376-19. doi: 10.1128/mBio.00376-19 (PMC6538776; doi:10.1128/mBio.00376-19)
Supplement: TEXT S1 [file mBio.00376-19-s0001.pdf]

Imaging

1. Microscopy and image analysis

For imaging the cells were immobilized on 1% agarose in water slabs on object glasses (1) and phase contrast and fluorescence microscopy images were obtained using a Nikon Eclipse Ti microscope equipped with a C11440-22CU Hamamatsu ORCA camera, an Intensilight HG 130W lamp and the NIS elements software (version 4.20.01). First a phase contrast image was taken through a CFI Plan Apochromat DM 100× oil objective, followed by a MatP-mCherry image using custom mCherry filter ex570/20, dic600LP, em605LP, a ZapB or ZapN image using GFP filter ex480/40, dic505, em535/50 and finally a DAPI image using filter ex360/40, dic400, em460/25. Images were analysed with Coli-Inspector supported by the ObjectJ plugin for ImageJ (version 1.49v) (2). Briefly, the length and diameter of more than 1200 individual cells were marked and analysed in the phase contrast images. Fluorescence and phase contrast images were aligned and fluorescence background was subtracted as described (2). The fluorescence of each cell was collected in a one-pixel wide bar with the length of the cell. A map of the diameter or the fluorescence localization and intensity was generated with the cells sorted according to increasing cell from left to right. Because cells were grown to steady state, the length of the cells can be directly correlated to the cell division cycle age. An age profile is created from all cell profiles in a map of a particular age range. They are first resampled to a normalized cell length of 100 data points, then averaged to a single plot using the macro Coli-Inspector-03s in ObjectJ (2). Concentration of the number of MatP molecules per cell and the number of molecules of MatP at midcell were determined as described (2). Calculation of the Pearson coefficient of the colocalization of MatP and GlpT was determined as described (3).

2. SIM sample preparation and imaging

Micron holes (1.1-1.4 µm) (4) were made with a micropillar mould in a 3% agarose in Gb4 medium layer to orient the cells vertically. Cells were concentrated by centrifugation and applied to the agarose alive. Part of the cells would enter the holes while another part would lay on top of the layer. To immobilize the cells in these holes, as MG1655 bacteria are able to rotate and move when imaged alive as they have flagella, a thin layer of 1% low melting point agarose in Gb4 medium was applied on top. A cover glass was then applied and taped to the glass slide.

SIM images were obtained with a Nikon Ti Eclipse microscope and captured using a Hamamatsu Orca-Flash 4.0 LT camera. Images were obtained with a SR APO TIRF 100x/1.49 oil objective, using 3D-SIM illumination with a 488 nm laser and an exposure time of 0.3 sec for the mNeonGreen-GlpT and a 561 nm laser with an exposure time of 1 sec for the MatP-mCherry, and were reconstructed (note that each reconstructed SIM image consists of 15 images) with Nikon-SIM software using for

each picture adapted values for the parameters Illumination Modulation Contrast (IMC), High Resolution Noise suppression (HNS) and Out of focus Blur Suppression (OBS).

Supplementary references

1. Koppelman CM, Aarsman ME, Postmus J, Pas E, Muijsers AO, Scheffers DJ, Nanninga N, den Blaauwen T. 2004. R174 of Escherichia coli FtsZ is involved in membrane interaction and protofilament bundling, and is essential for cell division. Mol Microbiol 51:645-57.
2. Vischer NO, Verheul J, Postma M, van den Berg van Saparoea B, Galli E, Natale P, Gerdes K, Luirink J, Vollmer W, Vicente M, den Blaauwen T. 2015. Cell age dependent concentration of Escherichia coli divisome proteins analyzed with ImageJ and ObjectJ. Front Microbiol 6:586.
3. Pende N, Wang J, Weber PM, Verheul J, Kuru E, Rittmann SKR, Leisch N, VanNieuwenhze MS, Brun YV, den Blaauwen T, Bulgheresi S. 2018. Host-Polarized Cell Growth in Animal Symbionts. Curr Biol 28:1039-1051 e5.
4. Söderström B, Chan H, Shilling PJ, Skoglund U, Daley DO. 2018. Spatial separation of FtsZ and FtsN during cell division. Mol Microbiol 107:387-401.
